# Enhancing cancer prevention and survivorship care with a videoconferencing model for continuing education: a mixed-methods study to identify barriers and incentives to participation

**DOI:** 10.1093/jamiaopen/ooac004

**Published:** 2022-02-12

**Authors:** Zheng Z Milgrom, Tyler S Severance, Caitlin M Scanlon, Anyé T Carson, Andrea D Janota, John L Burns, Terry A Vik, Joan M Duwve, Brian E Dixon, Eneida A Mendonca

**Affiliations:** 1 Center for Biomedical Informatics, Regenstrief Institute, Indianapolis, Indiana, USA; 2 Department of Epidemiology, Richard M. Fairbanks School of Public Health, Indiana University, Indianapolis, Indiana, USA; 3 Division of Pediatric Hematology Oncology, Riley Hospital for Children, Indianapolis, Indiana, USA; 4 Department of Pediatrics, Indiana University School of Medicine, Indianapolis, Indiana, USA; 5 Division Palliative Care, Riley Hospital for Children, Indianapolis, Indiana, USA; 6 Dean’s Office, Richard M. Fairbanks School of Public Health, Indiana University, Indianapolis, Indiana, USA; 7 Department of Radiology and Imaging Sciences, Indiana University School of Medicine, Indianapolis, Indiana, USA; 8 Kansas Department of Health and Environment, Topeka, Kansas, USA

**Keywords:** telemedicine, public health informatics, implementation science, education, continuing, cancer control

## Abstract

**Objective:**

To enhance cancer prevention and survivorship care by local health care providers, a school of public health introduced an innovative telelearning continuing education program using the Extension for Community Healthcare Outcomes (ECHO) model. In ECHO’s hub and spoke structure, synchronous videoconferencing connects frontline health professionals at various locations (“spokes”) with experts at the facilitation center (“hub”). Sessions include experts’ didactic presentations and case discussions led by spoke site participants. The objective of this study was to gain a better understanding of the reasons individuals choose or decline to participate in the Cancer ECHO program and to identify incentives and barriers to doing so.

**Materials and methods:**

Study participants were recruited from the hub team, spoke site participants, and providers who attended another ECHO program but not this one. Participants chose to take a survey or be interviewed. The Consolidated Framework for Implementation Research guided qualitative data coding and analysis.

**Results:**

We conducted 22 semistructured interviews and collected 30 surveys. Incentives identified included the program’s high-quality design, supportive learning climate, and access to information. Barriers included a lack of external incentives to participate and limited time available. Participants wanted more adaptability in program timing to fit providers’ busy schedules.

**Conclusion:**

Although the merits of the Cancer ECHO program were widely acknowledged, adaptations to facilitate participation and emphasize the program’s benefits may help overcome barriers to attending. As the number of telelearning programs grows, the results of this study point to ways to expand participation and spread health benefits more widely.

## Lay Summary

Cancer remains the second leading cause of death in the United States, and new methods are needed to support health care professionals in providing both prevention and survivorship care for their patients. To that end, a school of public health introduced an innovative telelearning program to use technology to link frontline health professionals at their locations with experts at the school for regularly scheduled learning sessions. These sessions on a range of cancer prevention and survivorship care topics take place twice a month and feature presentation of current information and discussion of cases. After the program’s pilot year, this study sought input from both participants and nonparticipants to identify incentives and barriers to participation. Input was collected via interviews and a survey. Incentives to participate included the program’s high-quality design, supportive learning climate, and access to information. Barriers included a lack of external incentives to participate and limited time available. Participants also wanted more adaptability in program timing to fit busy health care provider schedules. The results will be used to expand and improve the program in the future with the aim of enhancing providers’ use of cancer prevention strategies and care for survivors.

## BACKGROUND AND SIGNIFICANCE

Although death rates from cancer have declined over the last 20 years, it remains the second leading cause of death in the United States.^1^ Those national statistics are mirrored in Indiana, which continues to fall below state goals in factors affecting the prevention, screening, and survivorship.^2^ To help address these deficiencies by providing targeted support and education to local health care providers, the Indiana University-Purdue University at Indianapolis (IUPUI) Richard M. Fairbanks School of Public Health, partnering with the Indiana Cancer Consortium and Indiana Department of Health, introduced in September 2019 an innovative telementoring continuing education (CE) program using the Extension for Community Healthcare Outcomes (ECHO) model. The University of New Mexico introduced the ECHO model in 2004 to expand Hepatitis C care to rural communities in New Mexico; since that success, the model has spread to more than 60 countries with nearly 1300 programs as of August 2021.^3^^,^^4^

ECHO programs operate on a wheel-like hub and spoke structure, using multipoint, synchronous videoconferencing tools to connect frontline health professionals at various locations (the “spokes”) with expert teams at the facilitation center (the “hub”).^5^^,^^6^ A typical ECHO program offers weekly or biweekly sessions that consist of hub expert-prepared didactic presentations and group discussion of cases provided by spoke site participants. The Fairbanks School established its Project ECHO to engage health professionals across Indiana and beyond in remote learning programs to help them provide best-practice care in their communities; participation earns CE credits and is at no cost to participants. In addition to the cancer program, Project ECHO at the time of our study offered programs on Hepatitis C, LGBTQ+ issues, HIV, and pain management (one on coronavirus disease 2019 [COVID-19] was added later).

The Cancer Screening, Prevention, and Survivorship ECHO program (Cancer ECHO) holds a 1.5-hour session twice a month which typically includes a 20-minute didactic presentation by hub members and a 1-hour case discussion led by spoke site participants in a virtual grand rounds style. The didactic content covers a broad spectrum of cancer-related topics, ranging from lifestyle and vaccination prevention measures to motivational interviewing and smoking cessation to postradiation oncology surveillance, and repeats so participants can enroll at any time during the year. Hub team members are from oncology and its subspecialties, primary care, social work, psychology, and public and community health. In the pilot year (2019–2020), 22 sessions were held, and 147 spoke site individuals participated; 16 of those were primary care providers (PCPs). Sessions had an average of 14.5 spoke participants each, with an average of 2.5 PCPs (17.2%).

As the Cancer ECHO program transitions from the pilot stage to wider adoption and a routine operation, we want to expand spoke site participation. The objective of this study was therefore to gain a better understanding of the reasons individuals choose or decline to participate in the Cancer ECHO program and to identify incentives and barriers to doing so. Prior studies of ECHO programs suggest the model has high feasibility and promising educational effectiveness in new contexts,^7–10^ but none have provided in-depth analyses of participants’ and nonparticipants’ perceptions that can aid in the expansion of a program.

## METHODS

### Participant selection

Participants for this mixed-methods study were recruited based on purposeful sampling (with approval of the IU Institutional Review Board: #2002472006). Recruitment emails were sent to three groups: (1) the hub team (including IUPUI Project ECHO leadership that oversees all ECHO programs); (2) spoke site members who attended at least one Cancer ECHO session and may or may not have attended other Project ECHO programs; and (3) providers who attended one or more of the other Project ECHO programs but did not participate in the Cancer ECHO despite being invited, so we designated them potential spoke participants. All participants were asked to choose whether they would complete a survey or participate in an interview. The nonhub participants were provided with compensation of $10 to $40.

### Data collection and setting

Both the survey and the interviews explored individuals’ reasons for participation or nonparticipation in the Cancer ECHO program and their perceptions of the program. The anonymous survey was administered on IU Qualtrics, a secure web-based survey tool (Supplementary Table S1). In this quantitative part of the study, survey responses were reported using counts and percentages. For the qualitative part of the study, we used semistructured interviews (Supplementary Table S2). The interviews took place electronically and by phone which typically ran between 20 and 30 minutes. All interviews were conducted by an independent researcher (ZM), were recorded, and were transcribed with NVivo machine-transcription services with manual audits for accuracy.

### Theoretical framework

To organize and analyze data from the interviews, we chose to use a well-established conceptual framework: the Consolidated Framework for Implementation Research (CFIR). The CFIR synthesizes constructs from multiple theories into a robust framework and has been found effective for guiding the successful implementation of programmatic innovations in numerous health care domains.^11–15^ This framework, first published in 2009 and since updated, features a list of constructs categorized into five domains (Innovation Characteristics, Outer Setting, Inner Setting, Individual Characteristics, and Process) and provides a way to systematically assess barriers and facilitators to either prepare for or assess the implementation of an innovation.^16^

### Data analysis

**Figure 1.**
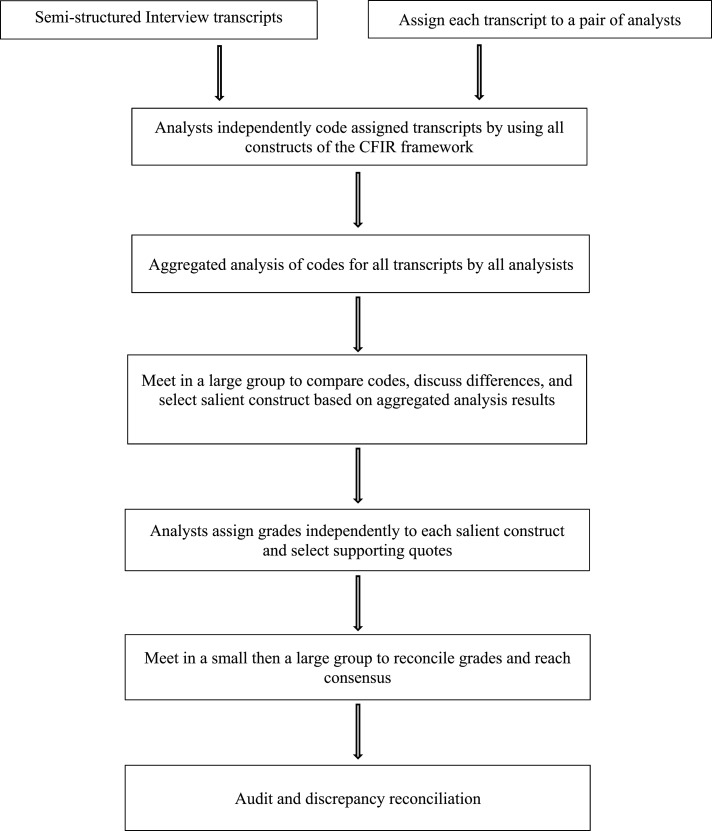
Workflow for coding and analysis of interview transcripts.

Three research team members (ZM, TS, and CS) independently coded the transcripts using CFIR constructs (Figure 1). Each transcript was coded by two analysts. Then, the three met as a group to discuss the aggregated analysis of codes, compare codes, discuss differences, and select the salient constructs from the full CFIR list of over three dozen. Salience was determined by measurable impact (positive or negative) of the construct on participation; nonsalient constructs appeared to have no impact on the decision to participate. Each analyst then assigned a score (−2, −1, 0, +1, +2, X) to each salient construct (Table 1). In group discussion, the analysts discussed their scores until reaching consensus. An auditor (EM) reviewed the process to increase the validity of findings and resolve any discrepancies. The coding and rating were guided by the CFIR codebook (http://www.wiki.cfirwiki.net/).

**Table 1.** Scoring scale for CFIR constructs identified as salient in interview transcripts

Score	Description
+2	At least two interviewees used explicit examples of the construct as having a positive impact on participation.
+1	The construct was mentioned or implied as having a positive impact on participation or was said to have a mixed but generally positive impact on participation.
0	The construct’s impact on participation was perceived to be neutral.
−1	The construct was mentioned or implied as having a negative impact on participation or was said to have a mixed but generally negative impact on participation.
−2	At least two interviewees used explicit examples of the construct as having a negative impact on participation.
X	The construct was perceived to have an equally positive and negative impact on participation.

## RESULTS

We conducted 22 semistructured interviews and received 30 anonymous surveys between May and September 2020. These participants’ gender, setting, and relationship to the program are shown in Table 2.

**Table 2.** Characteristics of interviewees and survey respondents

Characteristic	Interviews (*N* = 22)		Surveys (*N* = 30)	
	Number	Percentage	Number	Percentage
Gender				
Female	16	72.7	24	80.0
Male	6	27.3	6	20.0
Setting of practice				
Urban			14	46.7
Suburban or rural			11	36.7
Other/did not answer			5	16.6
Spokes: total	12		15	
PCP	5	41.7	4	26.7
Non-PCP	7	58.3	11	73.3
Attended other ECHOs	7	58.3	N/A	
Potential spokes: total	7		15	
PCP	6	85.7	10	66.7
Non-PCP	1	14.3	5	33.3
Attended other ECHOs	7	100	N/A	
Hub: total	3		0	
Provider	2	66.7		
Nonprovider	1	33.3		
Attended other ECHOs	2	66.7		

Spokes and potential spokes PCP: physicians, advanced practice nurses, and physician assistants.

Hub providers are specialist physicians and primary care providers. N/A: not applicable.

### Survey results

Among the survey respondents, 10–15% chose the program’s length, scheduled time, priority, or content as their primary reason for nonparticipation (Table 3). The respondents reported that the program’s length and scheduled time were most in need of change.

**Table 3.** Survey responses, by percentage of respondents

Response	Program length	Program time	Program not a priority for respondent	Program content	Survey responder plans to stay on track	Other reason/change
Primary reason for nonparticipation	15.2%	13.4%	10.9%	10.9%	10.9%	21.7%
Primary group/s with this reason	PS	All	S and PS	PS	H and S	All
Needs change	55.5%	59.8%	N/A	15.8%	N/A	
Primary group/s identifying this need	All	All		PS		
Primary change needed	32.0%	48.0%	N/A	4.0%	N/A	Preparation of case submission needs to be friendlier
Primary group/s identifying this need	All	All		PS		

PS: potential spoke participants; S: spoke participants; H: hub participants; N/A: not asked.

### Interview results

The consensus was that 13 of the CFIR constructs were salient influences on participation in the Cancer ECHO program. Six constructs had a positive impact, and six had a negative impact; one was equally positive and negative (Table 4). The following paragraphs present summaries of results for each salient construct, often defined by whether the interviewee was in our primary care provider (PCP) category (which included physicians, advanced practice nurses, and physician assistants) or was a non-PCP. Participants quoted in this section are designated by group (S, PS, H) and profession: physician (MD), family nurse practitioner (FNP), dentist (DDS), registered nurse (RN), certified health education specialist (CHES), master certified health education specialist (MCHES), and certified nurse assistant (CNA).

**Table 4.** CFIR constructs determined to be salient in interviews and consensus score assigned to each

Construct	Description	Score
I. Innovation characteristics		
Relative advantage	Stakeholders’ perception of advantage of implementing the intervention versus an alternative.	+2
Adaptability	Degree to which an intervention can be adapted, tailored, refined, or reinvented to meet local needs.	−1
Design quality and packaging	Perceived excellence in how the intervention is bundled, presented, and assembled.	+2
II. Outer setting		
External policy and incentives	Broad construct that includes external strategies to spread interventions, including policy and regulations, external mandates, recommendations and guidelines, pay-for-performance, collaboratives, and public or benchmark reporting.	−2
III. Inner setting		
Tension for change	Degree to which stakeholders perceive current situation as intolerable or needing change.	+2
Compatibility	Degree of fit between meaning and values attached to the intervention by involved individuals, how those align with individuals’ own norms, values, and perceived risks and needs, and how intervention fits with existing workflows and systems.	−1
Relative priority	Individuals’ shared perception of the importance of the implementation within the organization.	−1
Learning climate	A climate in which: (1) leaders express their own fallibility and need for team members’ assistance and input; (2) team members feel they are essential, valued, and knowledgeable partners in the change process; (3) individuals feel psychologically safe to try new methods; and (4) there is sufficient time and space for reflective thinking and evaluation.	+2
Available resources	Level of resources dedicated for implementation and ongoing operations, including money, training, education, physical space, and time.	−2
Access to knowledge and information	Ease of access to digestible information and knowledge about the intervention and how to incorporate it into work tasks.	+2
IV. Individual characteristics		
Knowledge and beliefs	Individuals’ attitudes about and value placed on the intervention as well as familiarity with facts and principles related to it.	X
V. Process		
Engaging	Attracting and involving appropriate individuals in implementation and use of the intervention through strategy of social marketing, education, role modeling, training, etc.	−1
Reflecting and evaluating	Quantitative and qualitative feedback about progress and quality of implementation accompanied with regular personal and team debriefing about progress and experience.	+2

*Note*: Definitions of constructs are adapted from CFIR Research Team, Center for Clinical Management Research. Consolidated Framework for Implementation Research (CFIR). https://cfirguide.org/constructs/. See Table 1 for definition of scores.

#### Relative advantage (+2)

Both PCP and non-PCP spoke and potential spoke interviewees spoke highly about the relative advantages of the ECHO model compared to other educational activities. They like that it is virtual, conversational, interactive, and interdisciplinary in nature. One interviewee (PS1, MD) said that, in other online continuing medical education (CME) programs, for “a lot of the activities you’re just in front of the computer screen doing some multiple choice or reading articles. I think [the ECHO program’s] real benefit is that with other people you have a community of support; you have feedback. So I feel like it can be a lot more fruitful than a lot of the other CME activities.”

#### Adaptability (−1)

The Cancer ECHO has a broad range of audiences, but some of the PCPs said they would prefer the program include further conversations within a smaller group or provide an asynchronous option to fit their schedules—suggesting they perceived it as not being adaptable to their needs. Current ECHO sessions are live-streamed with an option to replay a recording of the didactics part only but without asynchronous interactions. This construct refers only to the adaptability of the information exchange, not of the shared knowledge. This finding relates to the fact that the scheduled time was said in the interviews to be a major reason for nonparticipation and was the most selected aspect that needed to be changed on the survey (Table 3).

#### Design quality and packaging (+2)

The program assembled a dedicated hub panel who developed didactics topics with a holistic view of care. The hub team, with its broad range of expertise, also offered engagement and mentorship in the case-based learning, which enabled a collaborative model of education. The interviewees spoke highly of the program quality, especially the case discussion. “It’s been good to hear the [didactic] presentations, which is what I initially thought was [going to be] the most helpful,” said one interviewee (S6, FNP). “But actually, the case presentation and discussion component, where you have a question and answer, has been surprisingly more beneficial … at times and helps create change to my practice.”

#### External policy and incentives (−2)

This construct was especially important to PCPs. They considered the external policy and reimbursement for providing cancer control services at the point of care a significant negative factor for adopting the Cancer ECHO program and implementing its changes in their practices. Health professionals can earn CE credits by attending. However, not only potential spoke but also spoke PCPs expressed extensively that cancer control is a poorly incentivized field, which makes it a barrier for their participation in the program. One interviewee (S5, MD) noted, “I think one of the … major hassles that we find is that … the productivity requirements [for PCPs] are incredibly high…. You want to reach the agile clinicians, but you have to give them some kind of a carrot, and just increasing knowledge doesn’t do it.” Another (PS6, DDS) said, “It sounds bad, but people just come out and say that there’s no billable code for talking about smoking cessation.”

#### Tension for change (+2)

The interviewees agreed cancer control is an important topic that needs clear improvement, and they desired a change in the current performance of cancer control services. They mentioned many aspects impeding them from successfully delivering services to the needed population, such as insurance coverage and the difficulty of navigating the system and connecting to resources. “We have to improve cancer screening and prevention,” emphasized one interviewee (H2, MD). Another (S10, RN-CHES) said, “A lot of my burnout is also often related to system barriers and knowing our health care systems are very hard to navigate, even when you’re within the system.”

#### Compatibility (−1)

The interviewees, especially the potential spoke group, talked extensively about their patient population, the role of their specialty and services in cancer patient care, and the Cancer ECHO program’s perceived relevance and fit to their careers. Some said the pediatrics topics did not apply to their geriatric-dominant patient population. Others said they were not playing a big role in cancer survivorship care or were comfortable with their collaboration with specialists and felt the current content would be more meaningful to new care providers who do not yet have a niche in their practices. A dentist (PS 6, DDS) emphasized that he believes in dentists’ role in cancer control, but said that an unexpected conversation about cancer with patients “freaks them out sometimes.”

#### Relative priority (−1)

In general, spokes and potential spokes interviewees held a moderately negative attitude towards the relative priority of the program. “I think that I probably wouldn’t be able to allocate that much time [for the Cancer ECHO],” said one interviewee (PS4, MD). “The Hepatitis C [ECHO] is a little bit unique because … to be a primary care physician that prescribes medication for Hepatitis C, you’re legally required to attend the ECHO, so that sort of gives me and my organization a little bit more [incentive]… It’s a lot harder to kind of see that the value [of Cancer ECHO] adds to the cost of not seeing patients.” Another (PS7, MD) noted that, as busy care providers with many competing tasks, their choice of how to spend learning time “probably would be very topic-based.” On the survey, 10.9% of respondents reported the program’s not being a priority was their primary reason for nonparticipation. Asynchronous participation was proposed by some as a possible solution.

#### Learning climate (+2)

The spokes interviewees regarded highly the learning climate in the ECHO program, and the potential spokes interviewees who knew about IU ECHO programs also had confidence in the Cancer ECHO’s learning climate. A spoke participant (S6, FNP) said, “I think it’s empowering to feel like you’re a part of a team that is very broad and very large, that we are all working towards mutual goals, even in different specialties, in different roles, with different clinical backgrounds and different educational backgrounds. So that’s really neat to learn from other providers in other parts of the country potentially.”

#### Available resources (−2)

Comments in this construct primarily addressed the Physical Space and Time subconstruct. Thanks to the multipoint videoconferencing technology utilized, program participants were able to join without limitation in physical space resources, even during the pandemic shutdown. However, all subgroups of interviewees and survey respondents (Table 2) perceived their limited time as a major barrier. They were concerned about both the timing and length of the sessions. One interviewee (PS7, MD) said, “The time is the biggest thing, the time of day… That is the number one biggest barrier to me actually being able to participate. And the particular day chosen, I mean, primary care doctors do not get a lunch break.” Another (S7, MCHES) noted, “It is an hour and a half commitment, which is pretty long.”

#### Access to knowledge and information (+2)

A multidisciplinary hub team acted as the knowledge and information source and was dedicated to live-streaming support during sessions. An online resource library was also provided for individuals to review session recordings, didactic materials, and other resources mentioned during the session. Both spoke and potential spoke interviewees saw this access as a positive motivator. “I love the fact that they have the resources that they apply,” said one (S8, CHES). Another (PSI, MD) commented, “If you have a situation that you’re worried about, you can bring that up to the panel and they can also do a session on that.”

#### Knowledge and beliefs (X)

This construct had mixed data. Interviewees said they believed they could gain cancer-related knowledge from participating in the program, but PCPs perceived that complex systemic issues are what prevent patients from receiving optimal cancer preventive care, not care providers’ awareness or knowledge of best practices. The potential spokes and some spokes PCPs expressed uncertain beliefs in improving the issues in cancer control by mentoring PCPs, given the lack of incentives and worsening productivity requirements for care providers as well as unaffordability of preventive services. One PCP said that improving the operational side of health services would be more effective, while others expressed their belief in the ECHO model. One interviewee (PS3, MD) who is a medical director in an underserved area said, “I’m not interested in what are the ideal or best practices. I’m interested in what can we do for people with the resources that are available to us right now.” Another (S11, MD) said, “I think ECHO is right on target. You have to start somewhere.” Although some survey participants reported the ECHO content contributed to nonparticipation, only a few thought content change was the primary change needed (Table 2).

#### Engaging (−1)

Engaging PCPs to participate and present cases were perceived as a challenge. Although spokes interviewees said that they benefited the most from case discussions, the richness and the topics of that part of the sessions were not advertised to the target audience. Since case discussion is led by spokes PCPs, engaging them to actively present cases was limited by a relatively low PCP participation, despite frequent email communications between the program and its spokes and target audiences. Hub interviewees in particular pointed out concerns with this. “I don’t think we are capturing primary care providers, physicians in our ECHO participants,” said one (H2, MD). Another (H2, MD) noted, “I think the thing that’s been the toughest challenge is that we’re having a tougher time getting spoke sites to find good cases to present.”

#### Reflecting and evaluating (+2)

The hub team and spokes participants reported thinking the pilot year of the Cancer ECHO program went very well overall in terms of content quality, progress, and organization, provided useful resources and knowledge, and held stimulating discussions. One interviewee (S1, CNA) summarized this perspective by saying, “The organization of it [Cancer ECHO] and the layout of it were really impressive.” Although the hub team had difficulty obtaining real-time feedback from postsession surveys, a one-time survey found that 100% of spoke survey respondents (*N* = 14) reported the program met or exceeded their expectations.^17^ In the future, the hub team agreed to work on engagement, adjust the timing and frequency of sessions, and narrow topic choices from the broad range of possibilities in cancer screening, prevention, and survivorship.

## DISCUSSION

###  

#### Incentives and barriers to participation in cancer ECHO program

In our results, the ECHO intervention demonstrated its strong relative advantage over other educational methods in its virtual, interactive, and interdisciplinary format and its inclusion of learners’ real-life cases. This teleconferencing technology-enabled consistent participation even during the COVID-19 pandemic shutdown.^18^ These advantages were in marked contrast to the main PCP-targeted tool for best practice dissemination and quality improvement: electronic health record (EHR) alerts.^19^ On the Data, Information, Knowledge, and Wisdom pyramid, EHR alerts provide information on the case’s context and attempt to transform guidelines into case-relevant knowledge.^20^ However, EHR alerts are still called “cookbook medicine” and have limited compliance due to their inability to capture complex contextual nuances.^21^ Knowledge is not merely passing instructions; rather, as Weinberger explains, “We get to knowledge—especially ‘actionable’ knowledge—by having desires and curiosity, through plotting and play, by being wrong more often than right, by talking with others and forming social bonds.”^22^ In ECHO programs, participants are able to present the whole picture of their clinical cases, immerse themselves in a positive learning climate, and receive insights from both peers and experts, helping them progress from information to actionable knowledge. As distinguished from telemedicine that stops when the expert’s decision support on a case is received, ECHO telementoring aims to make learners self-sufficient at the end of a training cycle. An ECHO cannot help progress on the DIKW pyramid without a critical building block, PCPs, to provide contextual input and present and share insights for collaborative learning.

Even when anticipated knowledge gains are high, the decision for busy health professionals to participate in a telelearning intervention is complex. Adoption of innovation in healthcare generally involves multiple steps: acquaintance, persuasion, decision, initial adoption, and diffusion.^23^^,^^24^ In the first step, the target audience must become acquainted with the innovation. In our study, by recruiting from those who had various levels of experience with the Cancer ECHO, we were able to elicit knowledgeable opinions on factors impacting later stages of the adoption process. Our study found that one of the strongest barriers to PCPs’ deciding to participate in the program was available time. Other, though weaker, barriers were concerns about adaptability and compatibility of the program with providers’ needs and the fact that it was a low priority for many.

The other strongest barrier to participation in the Cancer ECHO program was the lack of favorable external policies and incentives in cancer control, which was discussed extensively by the interviewees and could be the upstream factor to other negative factors. Some interviewees compared external incentives for the cancer program with those for the Hepatitis C ECHO program, noting that filling a knowledge gap in Hepatitis C care results in physicians’ newly acquired capability and service expansion to treat those patients. Indeed, that program is required for PCPs in Indiana to receive Medicaid reimbursement for treating patients with Hepatitis C. Perhaps as a result, although overall participation in the Hepatitis C program was less than half that in the cancer program, PCP participation was much higher (54% vs 11% of total participants; Supplementary Figure S1). With the identification of all these barriers, improving the communication of the program’s perceived benefits to PCPs and fitting it to their needs for flexible participation and timing, including possibly exploring asynchronous options, became key goals for the program’s next stage.

A common issue of telehealth, poor context information-sharing,^25–27^ seemed to manifest as participation reluctance in our study. To encourage PCPs to commit to a program like this during the persuasion stage of the adoption process, they need information that thoroughly explains the context and highlights the program’s relevance and benefits to them. Information-sharing in the ECHO model functions in two ways: the hub manages and disseminates content and knowledge, while the spoke site participants provide cases for discussion and lead the case discussion during the session, with the support of the hub team. A key finding of our study is the disjuncture between the high value placed on the case discussions by the study participants, especially PCPs, and the barriers to their submitting cases. Not only does the submission of cases require extra time and effort on the part of PCPs, but the videoconferencing platform utilized by Project ECHO is separate from providers’ EHR, which are the most likely source of potential cases. The challenge of getting cases is not unique to our program: in a study of an ECHO program on tobacco cessation, 62% of respondents reported not having cases to present.^7^ Developing and streamlining methods to share privacy-protected case materials from EHRs with the ECHO programs would facilitate the submission of cases for discussion and boost this important aspect of the programs.

#### Strengths and limitations of the study

As the use of ECHO and other telehealth CE programs continues to grow, our study provides valuable insights into ways to both expand participation and enrich the experience. With cancer remaining the number two cause of death in the United States, there is a need for resources to help frontline health care providers expand prevention and survivorship care for their patients. Indeed, our study found strong agreement that new methods are needed in this area. Our results will be used to expand and improve our Cancer ECHO program, but they may also be helpful to other CE telelearning cancer prevention programs.

Our study also had limitations. It utilized retrospective data that was collected at the end of the pilot year, so results may have been affected by selective memory. Also, even with the use of both interviews and surveys, our sample was only a small part of the total population invited to participate in the study, and the results may not be representative of all Cancer ECHO program participants and potential participants.

## CONCLUSION

The Cancer ECHO program is at the intersection of telehealth technology, continuing professional education, and public health outreach. After its pilot year, this study sought to gain a better understanding of the reasons individuals choose or decline to participate in the program and to identify incentives and barriers to doing so. Incentives to participate included its high-quality design, supportive learning climate, and access to information. Barriers included a lack of external incentives to participate and limited time available. Participants also wanted more adaptability in program timing to fit health care providers’ busy schedules. These findings pointed to the need to make it easier to participate and to highlight the program’s relevance and benefits in order to overcome barriers to attending. There is a particular need to increase the participation of primary care providers and develop means to facilitate their submission of cases for discussion.

## FUNDING

This publication was made possible by a grant from the U.S. National Library of Medicine Grant number (T15LM012502). The Cancer Prevention, Survivorship and Prevention ECHO, a program of the IUPUI ECHO Center at the Indiana University Richard M. Fairbanks School of Public Health, described in this study received funding or in-kind support from the Indiana Department of Health (Contract #18938 “Cancer Prevention and Control Project ECHO plan”), specifically the Division of Chronic Disease, Primary Care, Rural Health, the Indiana Immunization Coalition (Contract #173640), Riley Children’s Hospital, American Cancer Society, Indiana Cancer Consortium, and the Indiana Clinical and Translational Sciences Institute. We also acknowledge the support from the Indiana Clinical and Translational Sciences Institute funded, in part by Grant Number (UL1TR002529) from the National Institutes of Health, National Center for Advancing Translational Sciences, Clinical and Translational Sciences Award. The content is solely the responsibility of the authors and does not necessarily represent the official views of the U.S. National Library of Medicine, the United States government, or any of the listed organizations or programs.

## AUTHOR CONTRIBUTIONS

All authors listed meet the ICMJE criteria for authorship for this manuscript. ZM, EM, JD, BD, AC, TS, and TV contributed to initial study design, and the development of protocol and interview and survey questions. All authors contributed to research strategy development. ZM collected the interview and survey data. EM, JD, BD, AC, and AJ supervised or assisted data collection. ZM, EM, TS, BD, TV, and JB contributed to the qualitative analysis strategies. ZM, TS, CS, and EM contributed to the qualitative analysis of coding and grade assignment. ZM, EM, TS, CS, JD, and BD contributed to the data interpretation. ZM wrote the initial draft, which was revised by all authors. All authors have approved the final manuscript to be published.

## SUPPLEMENTARY MATERIAL

Supplementary material is available at *JAMIA Open* online.

## Supplementary Material

ooac004_Supplementary_Data
