# Supplementary material for: Enhancing cancer prevention and survivorship care with a videoconferencing model for continuing education: a mixed-methods study to identify barriers and incentives to participation
Source: JAMIA Open. 2022 Feb 12;5(1):ooac004. doi: 10.1093/jamiaopen/ooac004 (PMC8846362; doi:10.1093/jamiaopen/ooac004)
Supplement: ooac004_Supplementary_Data [file ooac004_Supplementary_Data.docx]

**SUPPLEMENTAL ILLUSTRATIONS**

Supplemental Table 1. Survey

Supplemental Table 2. Semi-structured interview guide

Supplemental Figure 1. Total and primary care provider participants in Year 1 of five ECHO programs

Supplemental Table 1. Survey

Note: Only related contents are presented. A complete form of the survey instrument is available from the authors upon request.

Which of the following best describes your role in Cancer ECHO? (If you have an ECHO Record ID, you are enrolled in the Cancer ECHO program)

o Group 1. I did NOT enroll in Cancer ECHO, I was a GUEST in teleECHO clinics (1)

o Group 2. I did NOT enroll in or participate in Cancer ECHO, I ONLY heard about Cancer ECHO (I received Announcement/Recruitment emails, etc) (2)

o Group 3. I have ENROLLED in Cancer ECHO, but I have NEVER participated in any Cancer ECHO clinic session (3)

o Group 4. I am an ENROLLED 'Hub' member, and I have PARTICIPATED in Cancer ECHO clinic sessions (> =1 time) (4)

o Group 5. I am an ENROLLED 'Spoke' member, and I have PARTICIPATED in Cancer ECHO clinic sessions (>= 1 time) (5)

Continued question about your role in Cancer ECHO

o Group 4.1. I am a facilitator in the 'Hub' team (1)

o Group 4.2 I am a subject-matter in the 'Hub' team (2)

o Group 4.3 I am an administrative in the 'Hub' team (Program Director, Coordinator, etc.) (3)

o Group 4.4 I am playing other roles in the 'Hub' team (4)

o Group 5.1 I have PRESENTED a clinical case at a Cancer ECHO clinic (5)

o Group 5.2 I have NOT PRESENTED a clinical case at Cancer ECHO clinic (6)

o Group 5.3 I have attended Cancer ECHO, about 1 time/month or more (> = 50% of the sessions) (7)

o Group 5.4 I have attended Cancer ECHO, less than 1 time/month ( (8)

o (Group 1/2/3.1) I am a wrong target for Cancer ECHO, I do not have a chance to see patients with the needs of cancer prevention or survivorship care (9)

o (Group 1/2/3.2) I have a chance to see patients with the needs of cancer prevention or survivorship care (10)

o (Group 1/2/3.3) I am in the team that sees patients with the needs of cancer prevention or survivorship care, though I do not see them personally (11)

o Other (12)

What is your gender?

o Female (1)

o Male (2)

o Other (3) ________________________________________________

What is the type of your practice?

o Solo practice (1)

o Group practice (2)

o Health maintenance organization (HMO) (4)

o Hospital-based (5)

o Other (3) ________________________________________________

What is the setting of your practice?

o Urban (1)

o Suburban (2)

o Rural (3)

o Other (4) ________________________________________________

List 3 (or more) primary detailed reasons why you STOPPED attending the Cancer ECHO,

or why you attend/plan to attend LESS FREQUENTLY over time.

(Can select more than 1 answer, you can also select only 1 answer if it applies)

▢ I have been ATTENDING OVER 50% of the teleECHO clinic sessions (6 times or more before 3/4/2020) and am planning to stay on track (1)

▢ The time period (1st and 3rd Tuesday 12 pm to 1:30) IS the problem: "I have agenda conflicts during lunchtime" (2)

▢ The time period (1st and 3rd Tuesday 12 pm to 1:30)+ didactics ARE the problems: "I have other COMPETING tasks or Priorities during lunchtime, I only attend lunchtime sessions when the topics of the diacritics sound more relevant to my needs" (3)

▢ CASE PRESENTATION is the problem: "the focuses of the case discussions are LESS relevant to my practice" (4)

▢ A 1.5-hour session is TOO LONG (changing the LENGTH of clinic sessions WILL work) (5)

▢ The CONTENTS of Cancer ECHO, including the topics of diacritics and the focuses of case discussions, are overall LESS relevant to my practice: "I have been hesitated to invest 1.5 hours twice a month." (6)

If you are Group 1 or 2:

▢ I have technical issues (using Zoom) that cannot get addressed (7)

▢ I am only a trainee or not (yet) a practitioner, I am currently not committed to a full Cancer ECHO training (8)

▢ I am an interested practitioner, I would like to observe before I commit enrollment (9)

▢ Other Reason (10) ___________

What is the PRIMARY change, if we make, will INCREASE your likelihood of participation? (If we do not make this change, you will NOT be able to attend)

o Change the date from 1st and 3rd Tuesday each month, TO: (1) ____________

o Change the time of the day from 12 pm-1:30 pm, TO: (2) ___________

o Change the topic focuses of didactic, TO: (3) ____________

o Change the focuses of case discussion, TO: (4) ________________

o Change the length of each session from 1.5 hours, TO: (5) _____________

o The preparation of case presentation needs to be more presenter-friendly, such as: (6) _____

o Other (can be a COMBINATION of the above options): (7) ______________

o Nothing needs to be changed to keep me participating (8)

What are the suggestions that you wish us to make a change on? (If we do not make this change, you will be able to attend, but not as satisfied) (Can select more than one)

▢ Change the date from 1st and 3rd Tuesday each month, TO: (1) _____________

▢ Change the time of the day from 12 pm-1:30 pm, TO: (2) _____________

▢ Change the topic focuses of didactic, TO: (3) ___________

▢ Change the focuses of case discussion, TO: (4) ______________

▢ Change the length of each session from 1.5 hours, TO: (5) ___________

▢ The preparation of case presentation needs to be more presenter-friendly, such as: (6) _________

▢ Other honest suggestions: (7) __________

Supplemental Table 2. Semi-structured interview guide

| Dimension | Hub | Spokes | Potential Spokes |
| --- | --- | --- | --- |
| Characteristics of individuals | Can you tell me about yourself? What do you do at work? What is your role in the Cancer ECHO program? How many times did you attend the Cancer ECHO? | | |
| Motivation | - Why do you think people join the Cancer ECHO? | - Why did you decide to join the Cancer ECHO? | - What interested you when you heard about the Cancer ECHO? - How do you think this program can interest other practitioners to join? |
| Satisfaction | - Did the Cancer ECHO meet your expectations? Do you think it meets participants' expectations? | - Did the Cancer ECHO meet your expectations? In what way? | Not applicable |
|  | - Which session was your favorite and why? - Which session was your least favorite and why? | - Which session was your favorite and why? - Which session was your least favorite and why? | - Introduction of the program - Is there anything that you like about the curriculum? - Is there anything that you do not like about the curriculum? |
| Changes and beliefs | - How do you think the Cancer ECHO has changed the participants? | - Knowledge change? | - What are the challenges in cancer prevention and survivorship care? What are your needs to address those challenges? - Do you think the Cancer ECHO can change knowledge/confidence/practice/burnout levels of PCP? |
|  |  | - More confident in managing the complexities of cancer prevention and survivorship care? |  |
|  |  | - Professional practice? |  |
|  |  | - Emotional exhaustion change at work? - Sensitivity to my patients’ feelings change? - (Optional) Sense of personal accomplishment change from work? |  |
| Optional: Comparing to other continuing medical educations? |  | - How different the Cancer ECHO is comparing to other continuing medical education activities? | |
| Barriers | - What barriers do you think the participants have? | - Why did you stop attending/attend less frequently? | - Why did you never attend? |
| Suggestions | - What changes do you think the Cancer ECHO needs? - Other comments? | - What is the primary change that can increase your likelihood of participation? - Other comments? | |

Supplemental Figure 1. Total and primary care provider participants in Year 1 of five ECHO programs
